# The comparison of the efficacy of intramuscular tetracosactide and subacromial triamcinolone injection in rotator cuff tendinitis: a randomized trial

**DOI:** 10.1093/rap/rkae150

**Published:** 2024-12-20

**Authors:** Amir Hossein Orandi, Amirpasha Mansour, Nima Bagheri, Hossein Majedi, Seyed Ali Emami Meibodi, Seyed Khalil Pestehei, Peyman Saberian

**Affiliations:** Department of Anesthesiology, School of Medicine, Tehran University of Medical Sciences, Tehran, Iran; Department of Anesthesiology, School of Medicine, Tehran University of Medical Sciences, Tehran, Iran; Joint Reconstruction Research Center, Department of Orthopedics, Tehran University of Medical Sciences, Tehran, Iran; Pain Research Center, Neuroscience Institute, Imam Khomeini Hospital Complex, Tehran University of Medical Sciences, Tehran, Iran; Department of Anesthesiology, Critical Care and Pain Medicine, School of Medicine, Tehran University of Medical Sciences, Tehran, Iran; Pain Research Center, Neuroscience Institute, Imam Khomeini Hospital Complex, Tehran University of Medical Sciences, Tehran, Iran; Department of Anesthesiology, Critical Care and Pain Medicine, School of Medicine, Tehran University of Medical Sciences, Tehran, Iran; Department of Anesthesiology, Imam Khomeini Hospital Complex, Tehran University of Medical Sciences, Tehran, Iran; Department of Anesthesiology, Imam Khomeini Hospital Complex, Tehran University of Medical Sciences, Tehran, Iran

**Keywords:** rotator cuff tendinitis, ACTH, triamcinolone, tetracosactide

## Abstract

**Objectives:**

Rotator cuff tendinitis (RCT) is a tendon inflammation often following subacromial impingement syndrome. One of the non-surgical management modalities for RCT is subacromial injection of corticosteroids. Some studies have claimed a correlation between ACTH (Adrenocorticotropic Hormone) deficiency and rotator cuff lesions; hence, intramuscular ACTH analogue injection has been recommended as an option. This research aimed to compare the effectiveness of these two treatment methods.

**Methods:**

We conducted a study with 86 patients suffering from RCT. The patients were randomly divided into two groups of 43; one group received a subacromial injection of 40 mg of triamcinolone acetonide, while the other group received 1 mg of intramuscular tetracosactide injection. We recorded the Constant–Murley (CM) and visual analogue scale (VAS) scores for each patient before and 4 weeks after injections to measure pain acuity and joint functionality. Later, we compared and analysed the two scores in each group.

**Results:**

Based on the statistical analysis, the mean ages of the participants in the triamcinolone and tetracosactide groups were 53.21 ± 11.37 and 54.56 ± 11.98, respectively. Both groups demonstrated an improvement in VAS for pain and CM scores (*P* < 0.05). However, the VAS for pain score decreased, and the CM score increased more significantly in the triamcinolone group than in the tetracosactide group (*P* < 0.05).

**Conclusion:**

Although both treatment methods exhibit promise for pain relief, subacromial injection of triamcinolone appears more efficacious than intramuscular injection of tetracosactide in patients with RCT, based on a 4-week follow-up.

**Trial registration:**

Iranian Registry of Clinical Trials, https://irct.behdasht.gov.ir, IRCT20240110060673N1.

Key messages

- Our study found that intramuscular tetracosactide injections are effective in rotator cuff tendonitis.
- In comparison, triamcinolone injection is more effective than tetracosactide injection in rotator cuff tendinitis.
- Administering tetracosactide through an intramuscular injection is easier than performing a subacromial triamcinolone injection.

## Introduction 

The rotator cuff is a group of muscles and tendons stabilizing the shoulder joint. It includes the subscapularis, supraspinatus, infraspinatus, and teres minor muscles [1]. Shoulder pain, with a lifetime prevalence rate of 67%, is primarily attributed to subacromial impingement syndrome and rotator cuff pathologies. Rotator cuff injuries demonstrate an age-dependent distribution among patient populations. Prevalence rates escalate from 5% to 10% in patients under 20 and over 60% in patients aged 80 or older [2]. Rotator cuff tendinitis (RCT) is often along with subacromial impingement syndrome, which might happen following acute trauma or be a complication of chronic repetitive use or overuse of rotator cuff muscles during workouts or daily tasks [3, 4].

Management of RCT is divided into two categories: non-surgical techniques and surgical interventions. Treatment choice depends on factors such as age, physical activity, severity of symptoms, physical examination, and MRI findings [5]. As a non-surgical management, physical therapy is the first step. The following steps include isometric strengthening programmes, range of motion (ROM) exercises, prescription of NSAIDs (Non-steroidal anti-inflammatory drugs), and transcutaneous electrical nerve stimulations (TENS). One of the non-surgical palliative techniques is corticosteroid injection into the subacromial space. These methods also treat other musculoskeletal issues, such as knee pain [5–8].

Cosyntropin, or tetracosactide, is a synthetic derivative of adrenocorticotropic hormone (ACTH). It is mainly used for diagnostic purposes, but studies suggest the possible use of this drug for therapeutic purposes for patients unable to tolerate glucocorticoids or resistant to its effect [9–11]. It stimulates the melanocortin 2 (MC2) receptor, leading to full steroidogenic activity. When MC2 is activated in the adrenal cortex, it stimulates cortisol synthesis. These drugs, by inducing the production of corticosteroids, produce a glucocorticoid-dependent anti-inflammatory effect. However, tetracosactide also exerts anti-inflammatory effects through additional MC receptors, such as MC1, MC3, and MC5, commonly found on lymphocytes, neutrophils, macrophages, and endothelial cells. Stimulation of these receptors results in decreased cytokine production, reduced leucocyte infiltration, and enhanced phagocytosis [10].

In research conducted by Kim *et al.*, an association between ACTH deficiency and rotator cuff tear was established. This suggests that ACTH analogs may exert influence on tendinopathies not only through their anti-inflammatory effects but also through their effects on ACTH levels [10, 12].

This study compares two non-surgical methods for managing RCT: subacromial injection of corticosteroid (triamcinolone) and intramuscular injection of ACTH analogue (tetracosactide). The primary goal of this study is to provide valuable insights into the effectiveness of these treatments and contribute to the advancement of medical knowledge in this field.

## Methods

We designed the basic plan of this prospective, single-blind clinical trial to assess and compare the effects of subacromial injection of triamcinolone and intramuscular injection of tetracosactide in patients with RCT. This study enrolled patients diagnosed with chronic symptoms of RCT lasting over 6 months. Diagnoses were based on positive clinical examinations, specifically the Neer test and Hawkins–Kennedy test, performed by a single orthopaedic specialist. Additionally, the diagnoses were confirmed through ultrasonography (US) conducted by a single radiologist. A positive Neer test (Passive painful arc manoeuvre) is defined as feeling pain while passively flexing the glenohumeral joint while simultaneously preventing shoulder shrugging [13]. To perform the Hawkins–Kennedy test, one hand stabilizes the patient’s shoulder while the other rotates the shoulder internally, with the patient’s elbow flexed at 90 degrees. If the patient experiences pain during the test, it is considered a positive result [14]. For ultrasonographic findings, the guidelines of European Society of Musculoskeletal Radiology (ESSR) were used [15]. Each tendon was assessed via scanning planes in orientation as per longer axis, shorter axis, and by the myotendinous junction of shoulder to bony insertions and echoic changes or deformation in the tendon, including altered thickness without evidence of tear or non-continuity of the tendon fibres was accounted as RCT.

As per the recommendations outlined by the ESSR, when conducting sonographic examinations to locate the supraspinatus on the short axis, a healthy cuff should have a similar thickness extending 2 cm posteriorly from the biceps tendon landmark [15–17]. A GE Voluson E10 manufactured by the USA was used to assess US findings.

The exclusion criteria were a history of trauma or surgery on the shoulders, rheumatoid arthritis, fibromyalgia, frozen shoulder, drug abuse, low follow-up compliance (unable to revisit the doctor for follow-up), and synchronous shoulder pathology such as joint disease. After thoroughly explaining the treatment process to each patient, a consent form was provided, and upon signing it, the participants were included in the study. The past medical history and drug history of the patients were checked in terms of any interactions with tetracosactide or triamcinolone. The patients were randomly divided into two groups using computer-generated blocked-randomization numbers. Each patient took an envelope with a number in it, and based on that number, they were allocated. One group received a subacromial injection of 40 mg of triamcinolone acetonide (Cortiran 40 mg/ml, Iran Hormone, Tehran, Iran) performed by a single orthopaedic surgeon, while the other group received 1 mg of intramuscular tetracosactide (Synacran suspension for injection, 1 mg/1 ml, Iran Hormone, Tehran, Iran) injection performed by a single anaesthesiologist [10, 18].

For the subacromial injection of triamcinolone, the patients were asked to sit upright with their arms at their backs. Their shoulders were kept in an extended and internally rotated position. After sterilization of the region, using a high-resolution transducer (12 MHz linear array), a 3-ml syringe with a 21-gauge needle containing a mixture of 40 mg of triamcinolone acetonide and 2 ml of 1% lidocaine was injected into the subacromial bursa from the back of the shoulder [19, 20].

For intramuscular injection of tetracosactide, a 3-ml syringe with a 21-gauge needle containing 1 mg of intramuscular tetracosactide was inserted in the dorsogluteal region, 5–7.5 cm below the iliac crest in the upper outer quadrant of the butt at a 90-degree angle [21].

Patients were prohibited from using analgesics from 48 h prior to drug injection until 4 weeks post-injection to mitigate potential bias in the recorded outcomes. The study collected pain severity data from each patient in two stages: before the injection and 4 weeks after the injection. These data were obtained using the visual analogue scale (VAS) score. Patients were asked to put a mark on a scale ranging from 0 cm (no pain) to 10 cm (worst pain) to indicate their level of pain [22].

For better data analysis, VAS scores were divided into three categories: mild (scores of 3.4 cm or less), moderate (scores of 3.5–6.4), and severe (scores of 6.5 or more) [23]. The Constant–Murley (CM) score was also used to evaluate each case’s pain and functional ability in both stages (Table 1) [24]. The CM score is a tool used to assess four different aspects related to shoulder pathology. There are two subjective components, pain and activities of daily living (ADL), and two objective components, ROM and strength. The subjective components can receive up to 35 points, while the objective components can receive up to 65 points. This means that the maximum total score is 100 points, which indicates the best level of function. The patient answers the questions related to pain and ADL, while the ROM and strength are assessed through a physical examination [25].

**Table 1.** Constant–Murley score

Parameters	Degree (points)						
Pain	Severe 0		Moderate (5)	Mild (10)		None (15)	
Activities of daily living	Undisturbed sleep (2)		Complete recreational activity (4)		Complete daily work (4)		
Arm positioning	Above the head (10)	Up to the top of the head (8)	Up to the neck (6)	Up to the xiphoid (4)	Up to the waist (2)	Below the waist 0	
Range of motion	FF	0–30 (0)/31–60 (2)/61–90 (4)/91–120 (6)/121–150 (8)/≥151 (10)					0–40
	LE	0–30 0/31–60 (2)/61–90 (4)/91–120 (6)/121–150 (8)/≥151 (10)					
	ER	Hands behind head, elbows forward (+2) Hands behind head, elbows back (+2) Hands to the top of the head, elbows forward (+2) Hands to the top of the head, elbows back (+2) Full elevation of the arms (+2)					
	IR	Lateral aspect of the thigh (+0) Behind the buttock (+2) Sacroiliac joint (+4) Waist (+6) 12th thoracic vertebra (+8) Interscapular level (+10)					
Strength							0–25 Ibs
total							100

FF: forward flexion; LE: lateral elevation; ER: external rotation; IR: internal rotation.

The data were all recorded in the pre-prepared questionnaire. Finally, statistical analyses were performed using SPSS Statistics 26.0 (SPSS Inc., Chicago, IL, USA). Quantitative variables were presented as mean ± S.D., and qualitative variables were reported by rate and percentage. Statistical analyses were done using paired *t*-test, Mann–Whitney *U* test, two-sample independent *t*-test, and chi-square test. *P* < 0.05 was considered statistically significant for all analyses.

This clinical trial has received ethical approval from the Tehran University of Medical Science Ethics Committee under code IR.ARUMS.REC.1399.088 and has been registered in the Iranian Registry of Clinical Trial (IRCT20240110060673N1).

## Results

**Figure 1.**
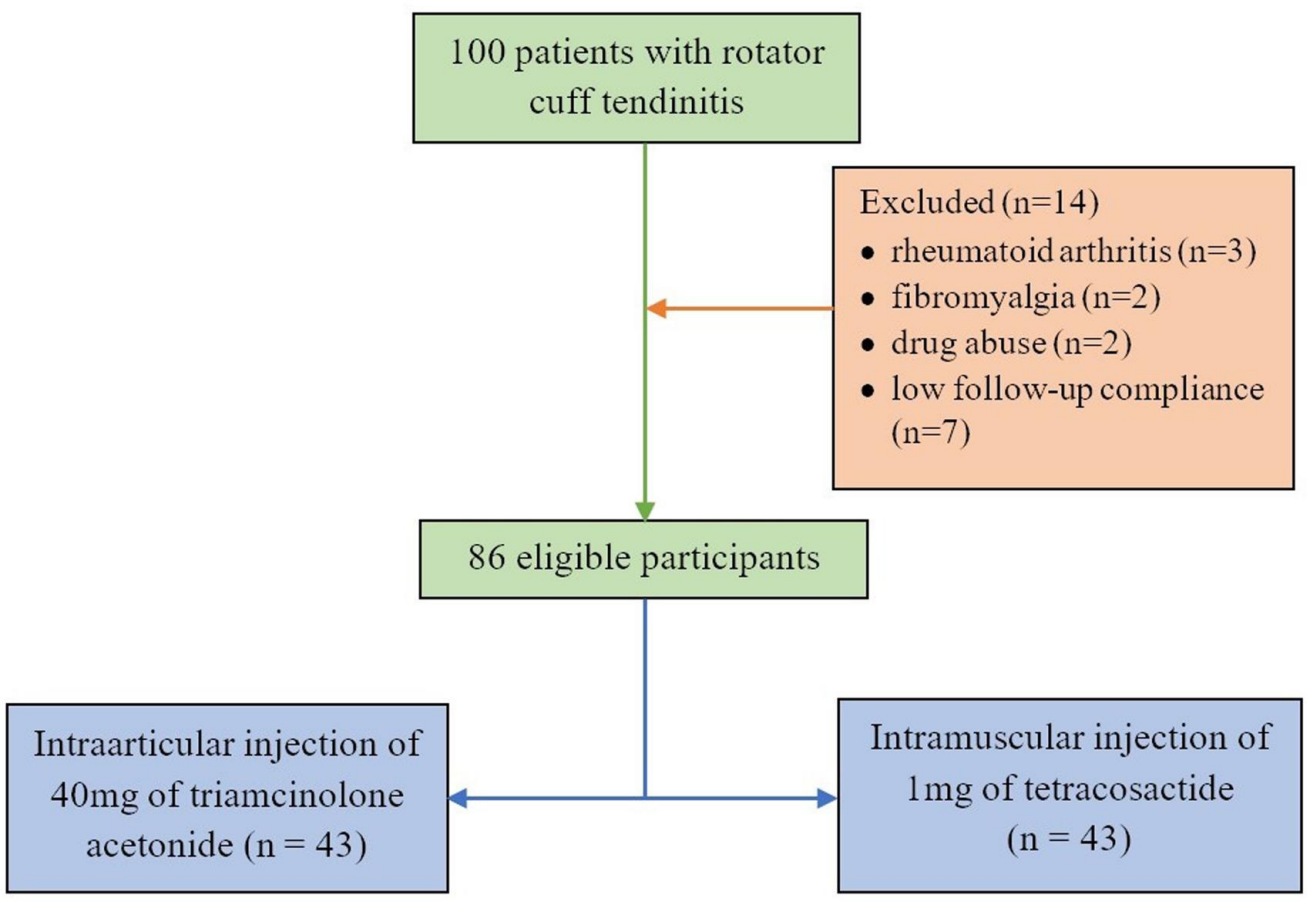
Study flowchart

Out of the initial group of 100 patients diagnosed with RCT, 14 were excluded from the study. Therefore, 86 eligible patients were enrolled in the study, with 43 patients in each group (Fig. 1).

The mean age of patients was 53.88 ± 11.63. Most participants, 39.5% (34), were male, and left shoulder involvement was more common with 45 patients (52.32%). A two-sample independent *t*-test showed that the two groups had no statistical difference in age (*P* > 0.05). The demographic results are shown in Table 2.

**Table 2.** The relationship between variables and study groups

Variables		Technique		*P*-value	95% CI Lower	95% CI Upper
		Triamcinolone	Tetracosactide			
Number of patients		43	43			
Age		53.21 ± 11.37	54.56 ± 11.98	0.594	−6.359	3.662
Gender	Male	14 (32.6%)	20 (46.5%)	0.186	−0.070	0.350
	Female	29 (67.4%)	23 (53.5%)			
Involved shoulder side	Right	22 (51.2%)	19 (44.2%)	0.517	−0.286	0.146
	Left	21 (48.8%)	24 (55.8%)			
VAS for pain before treatment	Mild	0 (0%)	3 (7%)	0.123	−0.051	0.423
	Moderate	13 (30.2%)	15 (34.9%)			
	Severe	30 (69.8%)	25 (58.1%)			
VAS for pain after treatment	Mild	19 (44.2%)	11 (25.6%)	**0.007**	**−**0.597	**−**0.100
	Moderate	24 (55.8%)	25 (58.1%)			
	Severe	0 (0%)	7 (16.3%)			

Two-sample independent *t*-test. *P*-values under 0.05 in bold.

VAS: visual analogue scale.

A two-sample independent *t*-test showed that both groups’ mean VAS scores for pain decreased following treatments. However, a higher reduction rate of VAS score for pain was achieved in the triamcinolone group compared with the tetracosactide group (*P* < 0.001 [95% CI 1.217–2.270]; Table 3).

**Table 3.** Comparison of VAS and CM scores between two groups before and after treatment

Variables	Triamcinolone	Tetracosactide	*P*-value	95% CI Lower	95% CI Upper
VAS for pain before treatment	7.19 ± 1.45	6.65 ± 1.95	0.153	−0.202	1.272
VAS for pain after treatment	3.42 ± 1.00	4.63 ± 1.66	**<0.001**	−0.1798	−0.620
Differences in VAS for pain	−3.76 ± 1.21	−2.02 ± 1.24	**<0.001**	1.217	2.270
CMS before treatment	49.77 ± 12.14	59.19 ± 15.07	**0.002**	−15.290	−3.543
CMS after treatment	63.49 ± 10.32	69.77 ± 13.09	**0.016**	−11.335	−1.223
Differences in CMS scores	13.72 ± 5.98	10.58 ± 5.36	**0.012**	0.701	5.577

Two-sample independent *t*-test. VAS: visual analogue scale; CMS: Constant–Murley score. *P*-values under 0.05 in bold.

**Figure 2.**
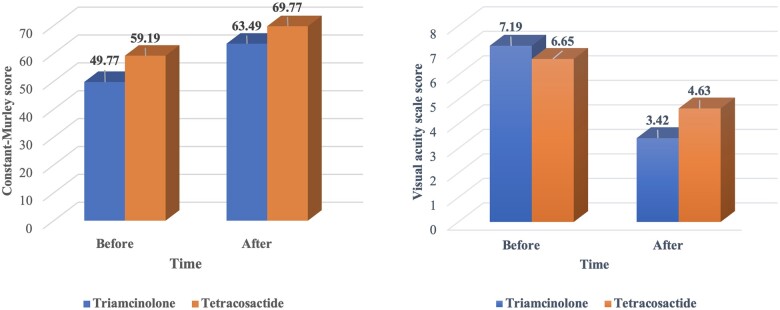
Comparison of median of CM score and VAS for pain between two groups before and after treatment

A paired *t*-test showed that the mean post-interventional VAS score for pain was significantly lower than the mean pre-interventional VAS score for pain in the triamcinolone and the tetracosactide group (*P* < 0.001 [95% CI 2.573–3.218]; Fig. 2).

Based on the Wilcoxon test comparison, the median of the post-interventional CM score was significantly higher than the median of the pre-interventional constant score in the triamcinolone group. The Mann–Whitney *U* test results also revealed that the rate of increase in CM score after intervention in the triamcinolone group was higher than in the tetracosactide (*P* < 0.05; Table 3, Fig. 2).

## Discussion

The shoulder joint is unique as it allows for a wide ROM in all directions. However, due to this extensive range, the shoulder is a relatively unstable joint and relies on soft-tissue structures to maintain its stability and function. If these soft-tissue structures are damaged, it can result in various shoulder problems. Shoulder pain is the second most common musculoskeletal complaint, after knee problems, for which patients seek medical help. It is estimated that between 7% and 34% of the population will suffer from shoulder pain at some point in their life. The most common cause of shoulder pain is rotator cuff lesions, which account for more than 70% of cases [25–27]. Hence, developing an effective management strategy for controlling shoulder pain is essential.

There are uncertainties regarding the effects of subacromial steroid injections in managing rotator cuff lesions. Some studies suggest that these injections work by reducing inflammation in the area, while others argue that the nature of these lesions is more degenerative than inflammatory [28, 29]. These findings further highlight the need for caution when considering the use of corticosteroids in treating tendinopathy due to the relationship between excessive cortisol and tendinopathy, its short-term effects, and inhibition of collagen synthesis, which interferes with the tendon healing process in some cases, such as acute tendinitis [27, 29, 30]. Contrary to the studies mentioned earlier, researchers like Akgün *et al*. have examined the advantages of local subacromial corticosteroid injection in subacromial impingement syndrome and recommend using corticosteroid injections in the acute and subacute phases of RCT treatment in addition to NSAIDs [31]. Overall, the peritendinous injection of corticosteroids in cases of RCT could have beneficial effects with its anti-inflammatory effects and may inhibit the production of collagen, other extracellular matrix molecules, and granulation tissue, resulting in improved connective tissue, peritendinous adhesions, and provide pain control [27]. Our study indicates that the group treated with triamcinolone injection experienced a significant reduction in pain in 4 weeks of follow-up. Moreover, when comparing the CM scores, we found that the triamcinolone group also exhibited an improvement in shoulder function.

Although studies suggest that a relationship between hypercortisolism and shoulder pain may exist [32], it has been suggested that a temporary deficiency of ACTH may contribute to a variety of shoulder lesions, including conditions such as RCTs and frozen shoulder [33, 34]. Additionally, some researchers have studied the potential therapeutic benefits of ACTH analogues for treating various shoulder conditions, such as acute subacromial bursitis [34].

Quigley and Renold assessed the effectiveness of an ACTH analogue for treating RCT and frozen shoulder. Their findings revealed that ACTH injection reduced local inflammation and decreased oedema and exudate in the medium term [35]. In our follow-up study, the pain and disability of patients in the tetracosactide group were relieved during 4 weeks; however, the relief was less than that of the triamcinolone group. It is worth noting that while our study results are consistent with one another, Quigley and Renold conducted an interventional trial involving a small population and did not compare their findings with any other therapeutic methods. In contrast, our study utilized a comparative design with a larger sample size, making our results more practical than those of Quigley and Renold.

None of the mentioned studies were similar to ours in terms of comparative methodology. After extensive research, we found only one study with the same methodology as our subject, conducted by Graham *et al*. in 1952. This study compared subacromial steroid injections with intramuscular ACTH analogues for treating rotator cuff pain. Unfortunately, the results of this research are not available [36]. Since then, no other studies have performed comparative trials.

Both treatment methods showed positive results during 4 weeks. However, the triamcinolone injection provided a more significant improvement in pain and shoulder functionality when compared with the ACTH analogue. This finding may result in using ACTH analogs in cases where corticosteroids are not recommended, such as in acute tendinitis, diabetes mellitus, or when the performer is inexperienced, and there is a risk of intratendinous injection [29, 30, 37].

One of the limitations of this study was that we focused on evaluating pain intensity and joint function in groups and did not assess the treatment’s adverse effects. The precision of the study would have improved if patients were followed up for at least 6 months. Although our exclusion criteria helped prevent bias, studying comorbidities would have also been beneficial. Additionally, understanding the efficacy of these methods would improve if these two groups were compared with a group receiving a placebo or not receiving any treatment. Another limitation of our study was that despite the strict restrictions on the use of analgesics by the patients, these drugs might have been used, which could have biased our data. Nevertheless, we took measures to prevent this by thoroughly explaining the study’s process and purpose. Also, measuring the levels of ACTH in patients would provide valuable insights into the relationship between ACTH levels and the development of tendinopathy. Furthermore, it would enhance our understanding of the potential mechanism underlying the impact of ACTH analogs through the elevation of ACTH levels. Future studies should compare the adverse effects of using ACTH analogs and corticosteroids for rotator cuff tendinopathies. However, it appears that a large sample of interventional and comparative trials with long-term follow-ups is necessary to arrive at a definite practical conclusion. In addition, in future studies investigating the effects of ACTH analogs on tendinopathies, it is crucial to measure ACTH levels in participants before and after injection.

Our 4-week follow-up has revealed that subacromial triamcinolone injection is more effective than intramuscular tetracosactide injection in rotator cuff pain relief and functional improvement. ACTH analogues offer a possible alternative when corticosteroids are not an option, especially considering the favourable outcomes of tetracosactide usage. One important aspect to consider is that an expert must perform the subacromial injection of triamcinolone, whereas the intramuscular injection of tetracosactide can be administered by a nonexpert individual. However, conducting extensive studies with long-term follow-up and MRI evaluation of shoulder joint alterations and therapeutic response is recommended to confirm our findings.

## Data Availability

The data generated and used in the study are available from the corresponding author upon reasonable request.
